# Identification of key enzalutamide-resistance-related genes in castration-resistant prostate cancer and verification of *RAD51* functions

**DOI:** 10.1515/med-2023-0715

**Published:** 2023-05-26

**Authors:** Wen Xu, Li Liu, Zhongqi Cui, Mingyang Li, Jinliang Ni, Nan Huang, Yue Zhang, Jie Luo, Limei Sun, Fenyong Sun

**Affiliations:** Shanghai Clinical College, Anhui Medical University, Shanghai, 200072, China; The Fifth School of Clinical Medicine, Anhui Medical University, Hefei, 230032, Anhui, China; Department of Clinical Laboratory Medicine, Shanghai Children’s Medical Center, School of Medicine, Shanghai Jiaotong University, Shanghai, 200127, China; Department of Clinical Laboratory, Shanghai Tenth People’s Hospital of Tongji University, 200072, Shanghai, China; School of Life Sciences, Jiangsu University, Zhenjiang, Jiangsu, 212013, China; Shanghai Clinical College, Anhui Medical University, No. 301, Yanchang Middle Road, Jingan District, Shanghai, 200072, China; Department of Clinical Laboratory, Shanghai Tenth People’s Hospital of Tongji University, No. 301, Yanchang Middle Road, Jingan District, 200072, Shanghai, China

**Keywords:** castration-resistant prostate cancer, prostate cancer, enzalutamide, *RAD51*

## Abstract

Patients with castration-resistant prostate cancer (CRPC) often develop drug resistance after treatment with enzalutamide. The goal of our study was to identify the key genes related to enzalutamide resistance in CRPC and to provide new gene targets for future research on improving the efficacy of enzalutamide. Differential expression genes (DEGs) associated with enzalutamide were obtained from the GSE151083 and GSE150807 datasets. We used R software, the DAVID database, protein–protein interaction networks, the Cytoscape program, and Gene Set Cancer Analysis for data analysis. The effect of *RAD51* knockdown on prostate cancer (PCa) cell lines was demonstrated using Cell Counting Kit-8, clone formation, and transwell migration experiments. Six hub genes with prognostic values were screened (*RAD51*, *BLM*, *DTL*, *RFC2*, *APOE*, and *EXO1*), which were significantly associated with immune cell infiltration in PCa. High *RAD51*, *BLM*, *EXO1*, and *RFC2* expression was associated with androgen receptor signaling pathway activation. Except for *APOE*, high expression of hub genes showed a significant negative correlation with the IC50 of Navitoclax and NPK76-II-72-1. *RAD51* knockdown inhibited the proliferation and migration of PC3 and DU145 cell lines and promoted apoptosis. Additionally, 22Rv1 cell proliferation was more significantly inhibited with *RAD51* knockdown than without *RAD51* knockdown under enzalutamide treatment. Overall, six key genes associated with enzalutamide resistance were screened (*RAD51*, *BLM*, *DTL*, *RFC2*, *APOE,* and *EXO1*), which are potential therapeutic targets for enzalutamide-resistant PCa in the future.

## 1 Introduction

According to Global Cancer Statistics 2020, prostate cancer (PCa) is a common cancer type that causes death worldwide in men. Approximately 1.4 million people are diagnosed with PCa worldwide, resulting in 375,000 deaths annually, which is an upward trend compared to 2018 [1,2]. The androgen receptor (AR) signaling pathway is involved in the entire process of PCa progression, including transformation and metastasis [3]. Therefore, androgen-deprivation therapy (ADT) constitutes the first-line therapy for PCa [4]. However, there are limitations to ADT therapy, and almost all patients treated with this method inevitably end up with castration-resistant prostate cancer (CRPC) [5,6].

Enzalutamide is an FDA-approved targeted drug for treating CRPC [7]. Enzalutamide is a small molecule oral agent that functions as an AR signaling inhibitor by negatively affecting AR nuclear translocation, thereby inhibiting the transcription of related drivers located downstream of the AR cascade. This results in the induction of apoptosis and suppression of CRPC cell proliferation [8,9]. Many studies demonstrate the positive effects of enzalutamide in CRPC patients, such as significantly prolonging survival after chemotherapy [10]. However, insensitivity to enzalutamide is found in almost half of the patients [11]. In addition, although some patients do better on initial treatment with enzalutamide, acquired resistance occurring after 11.2 months is common [12]. Given that the underlying mechanisms of enzalutamide resistance remain largely unclear, there is an urgent need to explore the causes of resistance to improve further therapeutic efficacy in CRPC.

Enzalutamide resistance in CRPC is a complex process that is usually associated with abnormal expression of certain genes, which play an important role in the development of enzalutamide resistance [13]. Researchers have actively explored genes associated with enzalutamide resistance. For example, AR-V7 is the most common variant detected in circulating tumor cells of patients with CRPC, which is significantly increased after enzalutamide treatment, and the splicing factors hnRNPA1 and Malat1 have been identified to mediate enzalutamide resistance by promoting the production and expression of AR-V7 [14]. Furthermore, galectin-3, a member of the animal lectin family, significantly inhibited the therapeutic effect of enzalutamide by increasing the expression of KLK3 and TMPRSS2 [15]. Although we have identified several genes associated with enzalutamide resistance, many unknown genes are likely to remain unidentified. Therefore, exploring aberrant genes associated with enzalutamide resistance will help discover biomarkers of resistance susceptibility and develop advanced therapeutic targets to provide more treatment options for patients with CRPC.

With the generation and application of high-throughput sequencing technologies, many important genes and signaling pathways involved in the cancer process have been massively mined and stored in public databases. Researchers can access and download the needed genomic data from international public knowledge databases such as Gene Expression Omnibus (GEO) and TCGA for free [16]. In the present study, we obtained data on enzalutamide-resistant and -sensitive cell lines from the GEO database and identified key genes involved in enzalutamide resistance through bioinformatics analysis. These results may provide new directions and new therapeutic targets for advancing research on CRPC resistance to enzalutamide.

## 2 Materials and methods

### 2.1 Date acquisition

Considering that the acquisition of drug resistance in enzalutamide-sensitive PCa cells is a slow process, we used cells cultured long term in enzalutamide (≥6 months) as a screening criterion to select the data we needed. We downloaded the high-throughput sequencing data of two different PCa cell lines treated with enzalutamide from the GEO database (GSE151083 and GSE150807 datasets; https://www.ncbi.nlm.nih.gov/geo/). GSE150807 contains data on enzalutamide-sensitive and -resistant LNCaP cell lines, while GSE151083 contains data on enzalutamide-sensitive and -resistant C42B cell lines. We also downloaded data related to prostate adenocarcinoma from TCGA database, which included 501 PCa and 52 normal samples. In addition, GSE32269, containing 22 primary PCa (hormone-dependent) and 29 metastatic CRPC tissues, was used for follow-up validation.

### 2.2 Analysis of differential expression genes (DEGs) associated with enzalutamide resistance

The “limma package” was used to analyze the DEGs in the GSE150807 and GSE151083 datasets. All DEGs between the enzalutamide-resistant and enzalutamide-sensitive cells were identified and visualized. To screen DEGs, |log2FoldChange| > 1 and adjusted *P*-value <0.05 were used as thresholds. The results were visualized in volcano and heat map plots, with the heat map showing only the top 20 highest and lowest expressed genes. Screened DEGs from the GSE151083 and GSE150807 datasets were intersected in a Venn diagram using Venny 2.1 (https://bioinfogp.cnb.csic.es/tools/venny/index.html), and overlapping DEGs common to both datasets were considered significantly associated with enzalutamide resistance.

### 2.3 Gene Ontology (GO) and Kyoto Encyclopedia of Genes and Genomes (KEGG) pathway analyses

To explore the potential biological functions of the DEGs associated with enzalutamide resistance, GO and KEGG analyses of the DEGs were investigated through the DAVID online website. The downloaded results were further analyzed using an online web tool (https://www.bioinformatics.com.cn). *P*-values <0.05 were considered statistically significant.

### 2.4 Construction of protein–protein interaction (PPI) network and identification of candidate hub genes

The PPI network for DEGs associated with enzalutamide resistance was built using STRING (STRING version 11.5). Interactions with a score >0.7 were considered statistically significant. The PPI network was visualized using Cytoscape software (v3.9.1), and the hub genes were determined using the plugin cytohubba in Cytoscape. The top 20 genes in the network, ranked using the maximal clique centrality (MCC) method, were treated as candidate hub genes.

### 2.5 Survival analysis and expression validation to identify hub genes

Gene Expression Profiling Interactive Analysis (GEPIA; http://gepia.cancer-pku.cn/), a versatile online analysis site based on TCGA and Genotype-Tissue Expression (GTEx) databases, was used to validate the correlation between the disease-free survival (DFS) and expression levels of the 20 candidate hub genes. Fragments per kilobase of exon per million mapped fragment (FPKM) data associated with PCa from TCGA database were downloaded, and we analyzed the expression differences of the 20 candidate hub genes between 501 PCa tissues and 52 normal tissues using the Wilcoxon test method. Finally, we visualized the results using the beeswarm package in R software. Through the above analysis, the candidate hub genes with clinical prognostic value were identified as hub genes. *P*-values < 0.05 were considered statistically significant.

### 2.6 Clinical parameter analysis of hub genes

The N- and T-stage-related information of the samples were extracted from the data related to PCa downloaded from TCGA, and the correlation between the expression levels of the hub genes and the N- and T-stage was investigated using the Wilcoxon test and Kruskal–Wallis test. *P*-values < 0.05 were considered statistically significant.

### 2.7 Analysis of the relationship between hub genes and enzalutamide

With enzalutamide being an AR signaling inhibitor, we evaluated the potential relationship between hub gene expression and the AR signaling pathway via Gene Set Cancer Analysis (GSCA; http://bioinfo.life.hust.edu.cn/GSCA/#/), a versatile platform for data analysis. Moreover, we analyzed the expression differences of the six hub genes between primary PCa and metastatic CRPC (mCRPC) tissue samples using Wilcoxon test based on the GSE32269 dataset. *P*-value < 0.05 was considered statistically significant.

### 2.8 Exploration of the potential relationship between hub genes and immune cell infiltration

The immune infiltration and mRNA expression module in GSCA was used to estimate the association between the expression levels of the hub genes and the infiltration levels of macrophages, monocytes, and natural killer (NK) cells. FDR < 0.05 was considered statistically significant.

### 2.9 Drug sensitivity evaluation

We determined the correlation between the expression of hub genes and drug IC50 via Pearson correlation analysis based on the Genomics of Drug Sensitivity in Cancer (GDSC) database through the drug sensitivity module in GSCA. This is done to identify potential molecular compounds for targeted therapies. FDR < 0.05 was considered statistically significant.

### 2.10 Cell line and culture

PCa cell lines PC3, DU145, and 22Rv1 were purchased from the American Type Culture Collection (ATCC). They were cultured in RPMI-1640 medium (Gibco, USA) containing 10% Fetal Bovine Serum (Ausbian, Cat. No. WS500T) and 1% penicillin/streptomycin (Gibco, USA) and maintained at 37°C in a 5% CO_2_ environment.

### 2.11 Transient transfection of cells

For transient transfection, 5 × 10^4^ cells/mL of PC3, DU145, and 22Rv1 cells were added to 6-well plates the day before transfection and cultured overnight. The next day, at about 30–40% confluence, the cells were transfected with Lipofectamine 2000 (Invitrogen, Carlsbad, CA, USA) and small interfering RNA (siRNA) and then further incubated for 48 h. The siRNAs targeting *RAD51* are shown in Table 1.

**Table 1:** Sequences of the two siRNAs

siRNA	siRNA sequence information
siRNA#1	5′-AAGCTATGTTCGCCATTAATT-3′
siRNA#2	5′-CGCCCTTTACAGAACAGACTA-3′

### 2.12 RNA extraction and quantitative reverse transcription-polymerase chain reaction (qRT-PCR)

The collected PC3, DU145, and 22Rv1 cell pellets were treated with TRIzol reagent (Invitrogen, Thermo Scientific, Shanghai, China) for total RNA extraction. The general process was as follows: cells were treated with TRIzol reagent for 10 min, centrifuged at 12,000 rpm for 15 min at 4°C, and then the upper aqueous phase was extracted and mixed with isopropyl alcohol for 10 min, and centrifuged for another 10 min under the same conditions. The supernatant was removed, the residual liquid was washed with absolute ethanol, and then centrifuged at 7,500 rpm for 5 min to remove the supernatant to obtain the RNA precipitate. Finally, the RNA precipitate was dissolved in RNase-free water, and RNA was quantified using Nano drop 300 (ALLSHENG). Total RNA was subsequently reverse transcribed to obtain cDNA using a reverse transcription kit (TaKaRa Bio, Shiga, Japan). Finally, the relative expression of *RAD51* was detected via qRT-PCR in a TK-6000 PCR system using a SYBR Green kit (TaKaRa Bio, Shiga, Japan). *GAPDH* expression was used for normalization of gene expression. Primers for qRT-PCR are listed in Table 2.

**Table 2:** qRT-PCR primer pairs

Name	Primer sequence information
RAD51-F	5′-CAACCCATTTCACGGTTAGAGC-3′
RAD51-R	5′-TTCTTTGGCGCATAGGCAACA-3′
GAPDH-F	5′-GGAGCGAGATCCCTCCAAAAT-3′
GAPDH-R	5′-GGCTGTTGTCATACTTCTCATGG-3′

### 2.13 Western blotting detection

PC3, DU145, and 22Rv1 cells were lysed with RIPA buffer containing protease and phosphatase inhibitors to isolate proteins. Following separation via a 10% sodium dodecyl-sulfate polyacrylamide gel electrophoresis, the protein samples (30 µg) were transferred to nitrocellulose membranes. The membranes were blocked with 5% non-fat powdered milk and incubated with rabbit antibody against RAD51 (Proteintech, Cat#: 14961-1-AP) and mouse antibody against Actin (Abmart, Cat#: M20011) for 1 h at 37°C, followed by washing of the membranes. The fluorescently conjugated secondary antibodies (Licor, USA) were incubated with the membrane for 1 h at room temperature. Protein signals were detected by a two-color infrared laser imaging system (Odyssey CLx). Actin protein expression was used as an internal control.

### 2.14 Cell counting kit (CCK)-8 and colony formation assays

PC3 and DU145 cells were treated with siRNA for 48 h, and the cell proliferation ability was determined using the CCK-8 kit (Share-bio, SB-CCK8). The colony formation ability of PCa cells was determined by colony formation assay [17].

### 2.15 Apoptosis assay

Transiently transfected PC3 and DU145 cells were collected in 1.5 mL EP tubes, and 50 μL of diluted fluorescein isothiocyanate (FITC) was added to each tube. After 20 min, 250 μL of propidium iodide was added for 5 min according to the instructions of the FITC Annexin V apoptosis Detection Kit (BD Biosciences, San Diego, CA). Finally, the samples were analyzed using the BD LSRFortessa analyzer (BD Biosciences).

### 2.16 Cell migration assay

The transwell migration assay was set up in 24-well plates containing 500 μL of medium containing 10% FBS. The transwell units were placed in the wells, and 400 μL of serum-free medium containing 5 × 10^4^ transiently transfected PC3 and DU145 cells was added to the corresponding upper chamber. The cells were incubated in a 5% CO_2_ incubator at 37°C for 24 h. Uninvaded cells on the membrane surface were gently removed using a wet swab, and the remaining cells were fixed with 4% paraformaldehyde for 15 min at room temperature before staining with 0.1% crystalline violet. Once the transwell units were dry, we examined them using an inverted microscope (Nikon). The invading cells were quantified using ImageJ software.

### 2.17 Drug treatments

RPMI-1640 medium containing 10% FBS and 1% penicillin/streptomycin was used to prepare 0, 5, 10, 20, 40, and 80 μM working concentrations of enzalutamide (Beyotime, Cat#: SC0074-10mM). The 22Rv1 cells siRNA-treated for 48 h were seeded at 1 × 10^4^ cells/well in 96-well plates. After 24 h, the medium in the wells was removed and 200 μL of enzalutamide medium was added to the wells according to the principle of five replicate wells for each concentration. Cell viability was determined by CCK-8 kit after 48 h treatment.

### 2.18 Statistical analysis

Statistical analysis of the data was performed using R software. Comparisons of two independent samples were analyzed using Wilcoxon test, and comparisons of multiple independent samples were analyzed using the Kruskal–Wallis test or ANOVA. *P*-value <0.05 was considered statistically significant. The relationships between gene expression and signaling pathway activity, drug sensitivity, and immune infiltration were automatically generated by an online website, and FDR < 0.05 was considered statistically significant.

**Figure 1:**
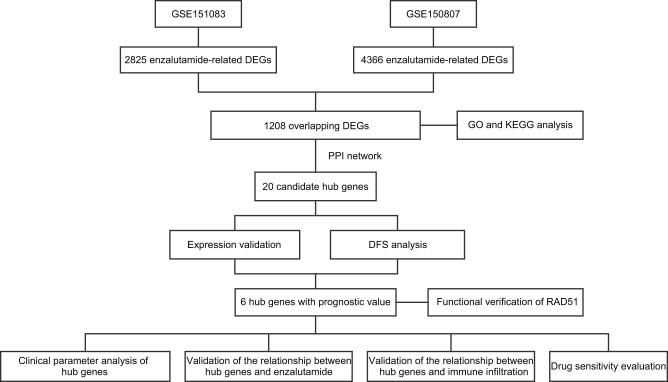
The design flow chart of this study. DEGs, differentially expressed genes; GO, Gene Ontology; KEGG, Kyoto Encyclopedia of Genes and Genomes; PPI, protein–protein interaction; DFS, disease-free survival.

## 3 Results

### 3.1 Identification of DEGs associated with enzalutamide resistance

**Figure 2:**
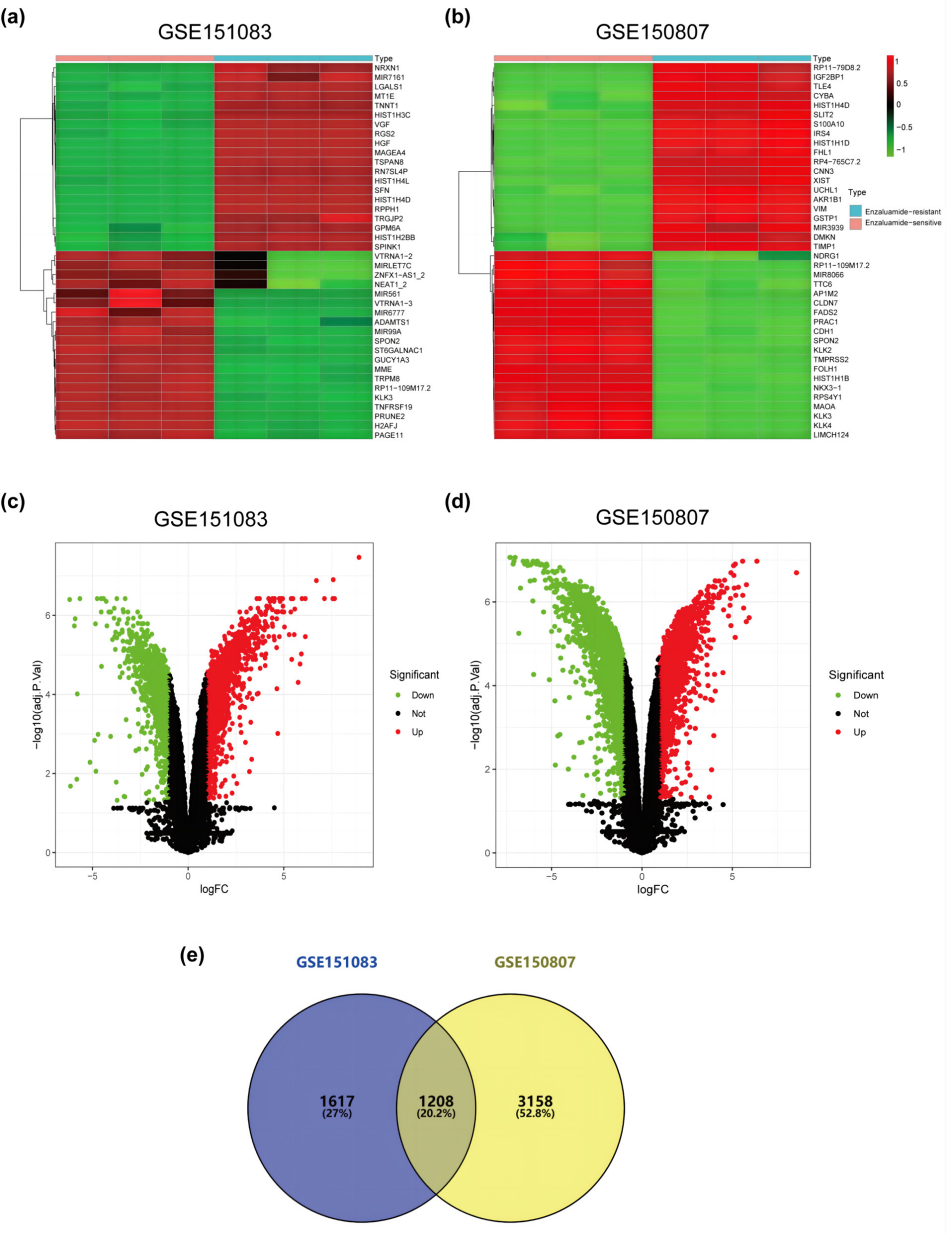
DEGs in enzalutamide-resistant and enzalutamide-sensitive PCa cell lines. (a and b) The heat map shows the top 20 DEGs in the GSE151083 and GSE150807 datasets with enzalutamide-resistant and enzalutamide-sensitive cell lines. (c and d) Volcano plot showing all DEGs in the GSE151083 and GSE150807 datasets with enzalutamide- resistant and enzalutamide-sensitive cell lines. (e) Venn diagram showing the overlapping DEGs in GSE151083 and GSE150807. DEGs, differentially expressed genes; PCa, prostate cancer.

Two GEO datasets were used to identify DEGs associated with enzalutamide resistance. After analysis, 4,366 and 2,825 differential genes were identified in GSE150807 and GSE151083, respectively (Figure 1). Volcano and heat maps were used to visualize these DEGs (Figure 2a–d), with the heat map showing only the top 20 highest and lowest expressed genes with the largest differential expression multiplicity. The Venn diagram shows 1,208 overlapping DEGs associated with enzalutamide resistance found in both datasets (Figure 2e).

### 3.2 GO and KEGG pathway analyses

**Figure 3:**
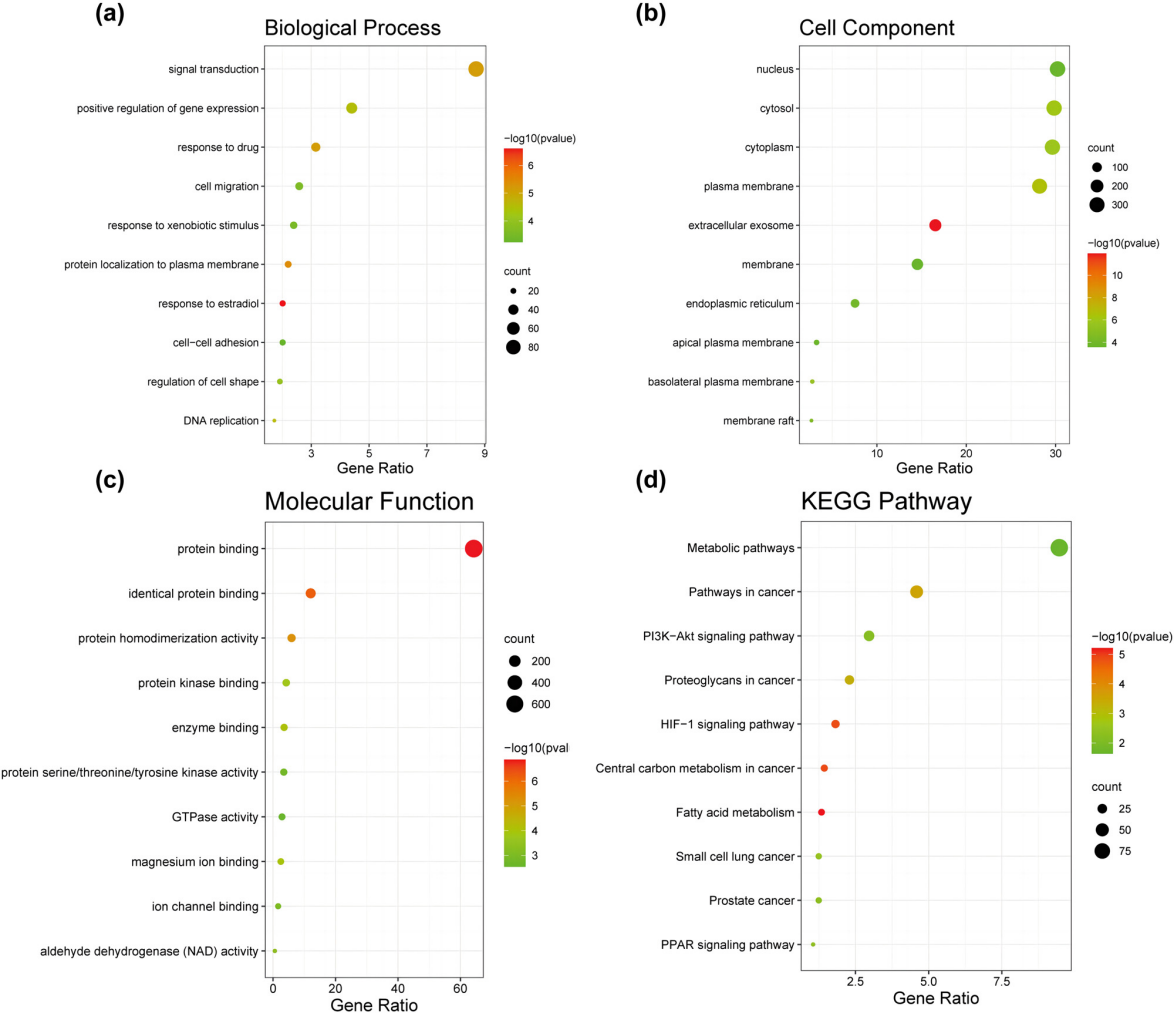
GO and KEGG analysis of DEGs. (a–d) Bubble plots showing the results of functional annotation analysis of 1,208 overlapping DEGs. (a) Biological process. (b) Cell component. (c) Molecular function. (d) KEGG pathways. GO, Gene Ontology; KEGG, Kyoto Encyclopedia of Genes and Genomes; DEGs, differentially expressed genes.

To further explore the potential functions of DEGs, we performed GO and KEGG analysis. As shown in Figure 3, biological process analysis showed that DEGs were mainly enriched in signal transduction, positive regulation of gene expression, and drug response. Cellular component analysis showed that the nucleus, cytosol, cytoplasm, and plasma membrane were the most common classifications. For the molecular function, protein binding was significantly enriched. In addition, KEGG pathway analysis showed that these genes were involved in metabolic pathways and cancer-related pathways.

### 3.3 Construction of PPI network and identification of candidate hub genes

**Figure 4:**
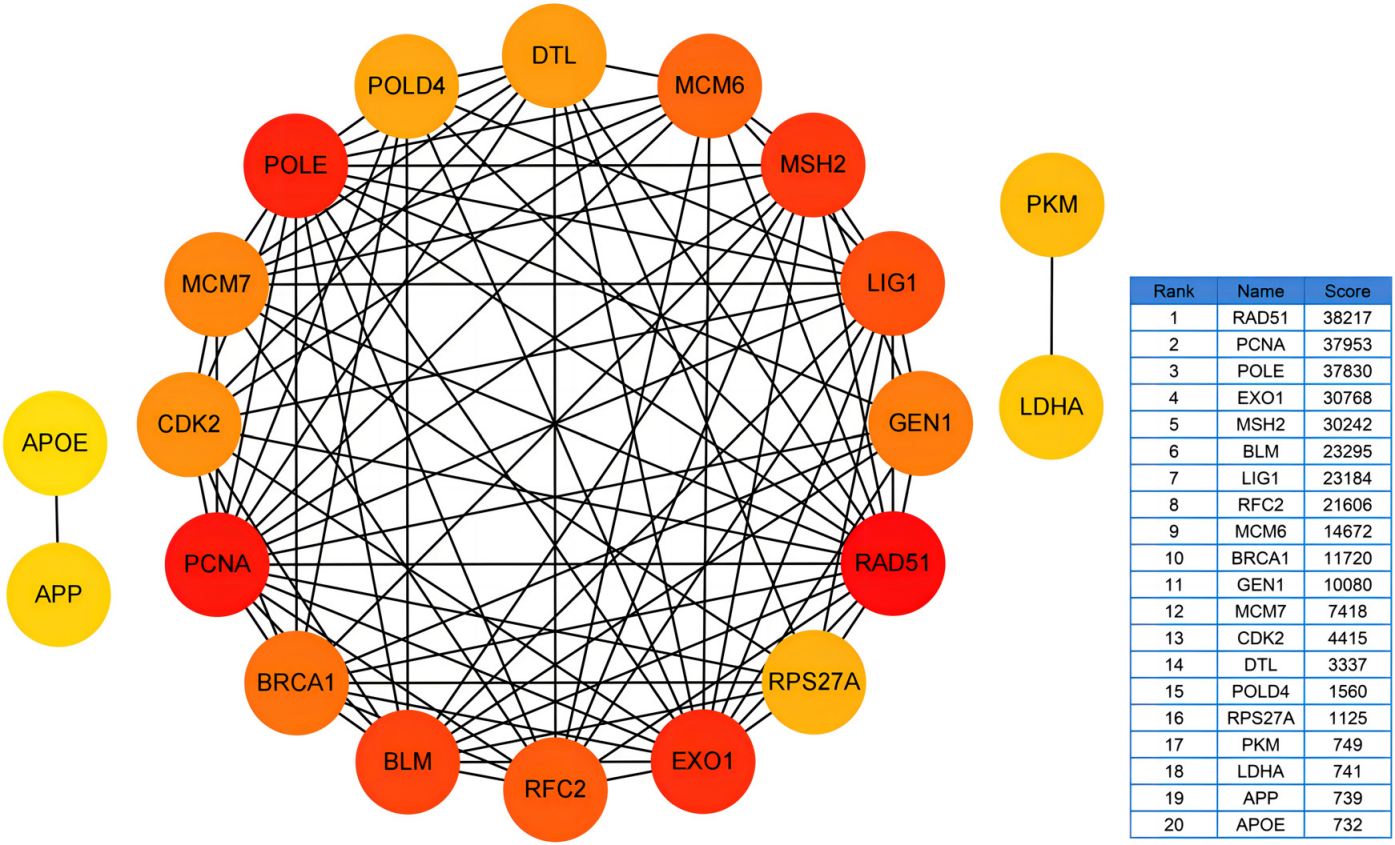
The PPI network shows the top 20 genes with the highest degree of connectivity calculated by the MCC algorithm in cytohubba from 1,208 overlapping genes. PPI, protein–protein interaction network; MCC, maximal clique centrality.

We uploaded 1,208 overlapping DEGs to the STRING database and the analyzed data were downloaded. The PPI network was then imported into Cytoscope and the top 20 genes with the highest degree of connectivity were derived by the MCC algorithm using the plugin cytohubba (Figure 4). These 20 genes – *RAD51*, *PCNA*, *POLE*, *EXO1*, *MSH2*, *BLM*, *LIG1*, *RFC2*, *MCM6*, *BRCA1*, *GEN1*, *MCM7*, *CDK2*, *DTL*, *POLD4*, *RPS27A*, *PKM*, *LDHA*, *APP*, and Apolipoprotein E (*APOE*) – were identified as candidate hub genes.

### 3.4 Identification of hub genes through expression and survival analyses of candidate genes

**Figure 5:**
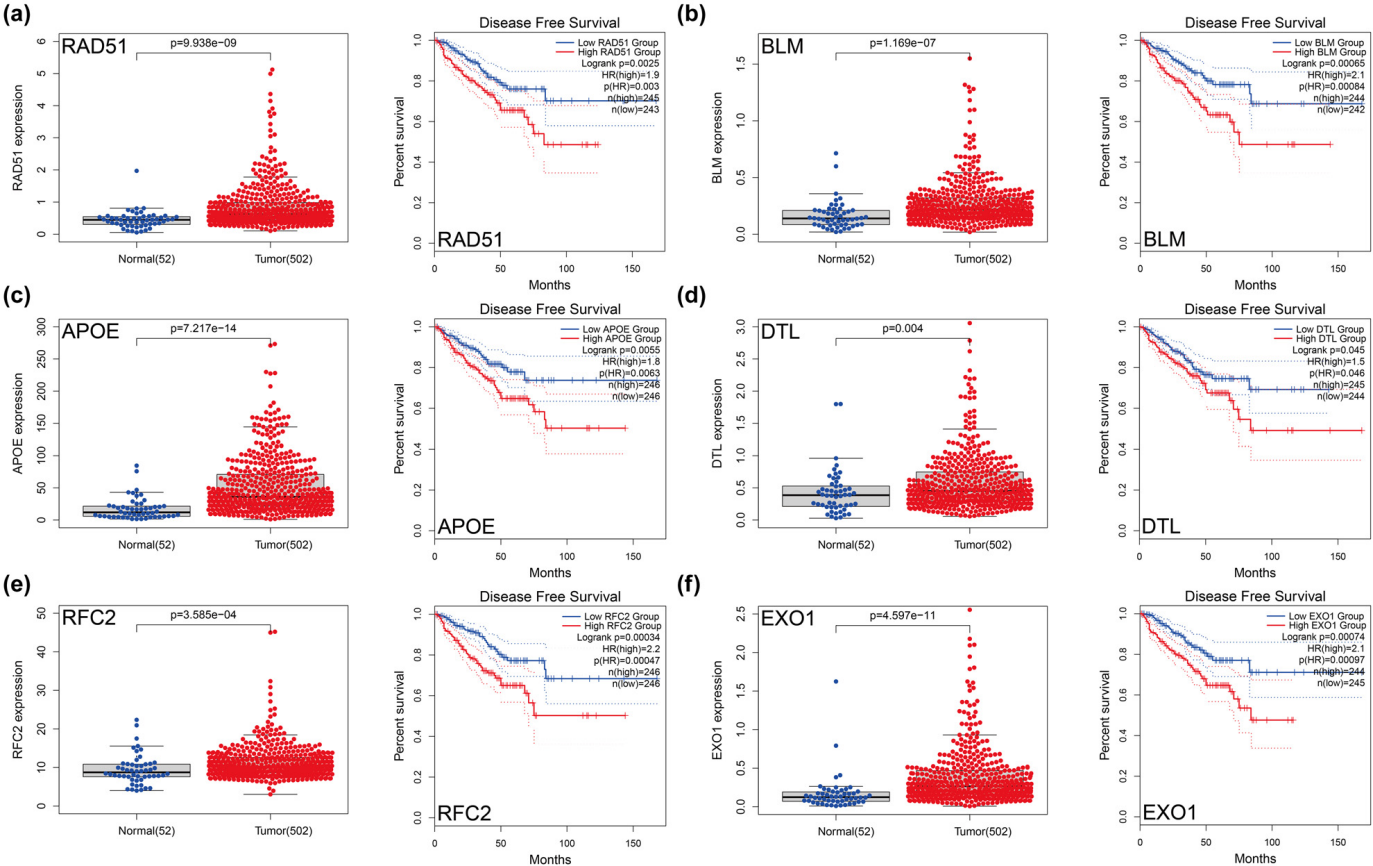
Validation of the expression and DFS of top 6 genes. (a) RAD51. (b) BLM. (c) APOE. (d) DTL. (e) RFC2. (f) EXO1. The significance of differences between the two groups was determined using the Wilcoxon test. DFS, disease-free survival.

To screen for hub genes with clinical application, we explored the differences in expression of candidate hub genes in PCa and normal tissues, including DFS. We identified six genes, including *RAD51*, *BLM*, *APOE*, *DTL*, *RFC2*, and *EXO1*, with high expression associated with poorer DFS (Figure 5). These six genes with prognostic value were defined as hub genes.

### 3.5 Clinical parameter analysis of hub genes

**Figure 6:**
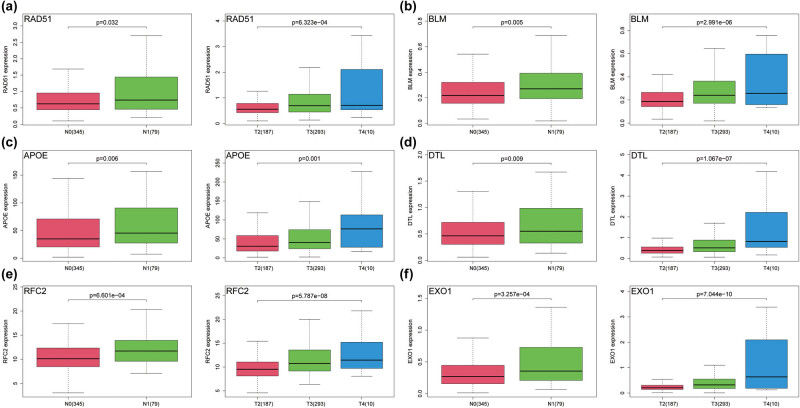
Validation of the correlation between the expression levels of hub genes and N-stage and T-stage. (a) RAD51. (b) BLM. (c) APOE. (d) DTL. (e) RFC2. (f) EXO1. The significance of differences between the two groups was determined using the Wilcoxon test, and Kruskal–Wallis test was used to compare multiple groups.

In order to explore whether the hub genes had clinical value, we focused on the relationship between their expression level and N- and T-stages. Our results showed that the expression levels of these six hub genes increased with the increase in N- and T-stages of PCa (*P* < 0.05; Figure 6).

### 3.6 Validation of the relationship between hub genes and enzalutamide

**Figure 7:**
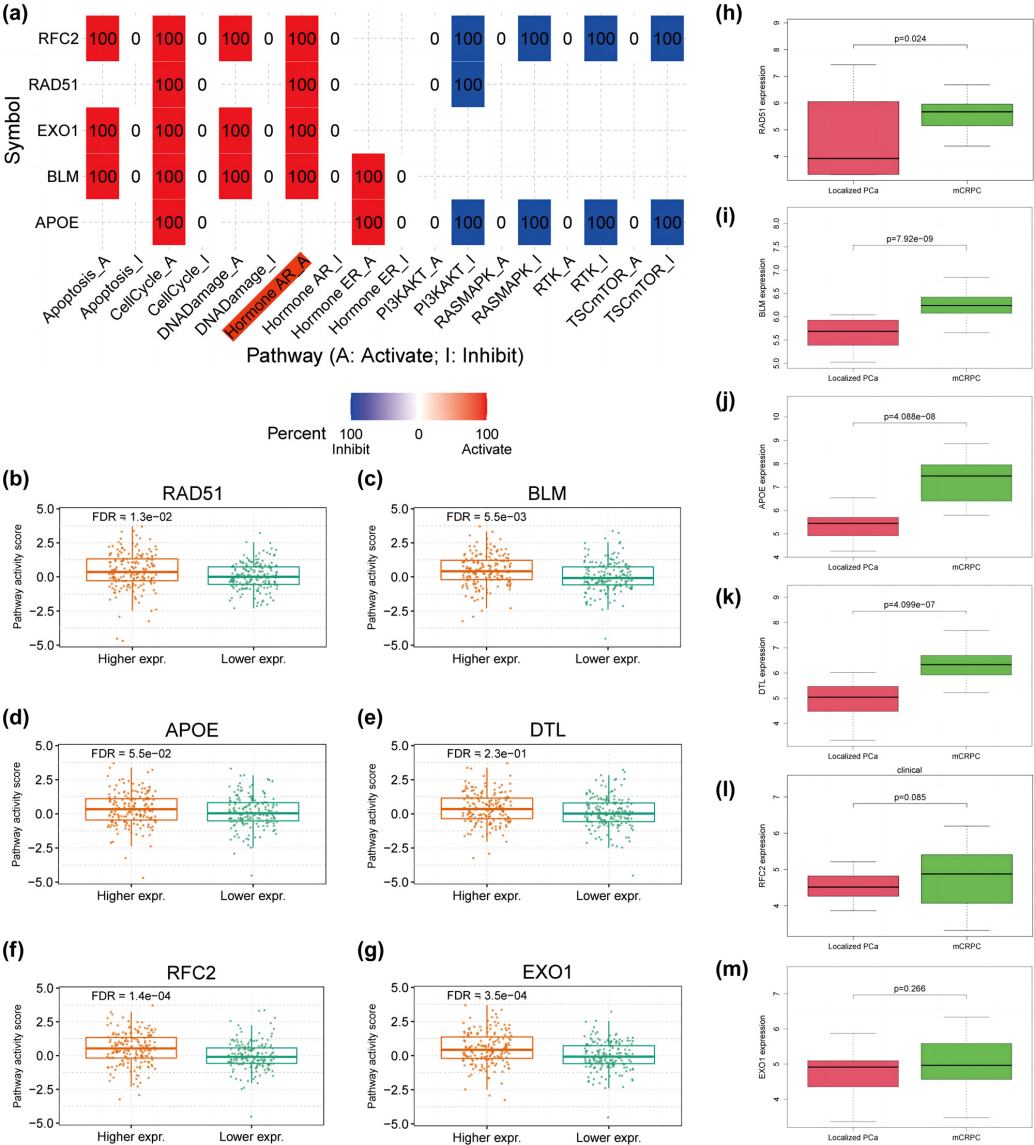
Validation of the relationship between the expression levels of hub genes and the AR signaling pathway. (a) The potential role of hub genes on signaling pathways in PCa is summarized in the figure. The red module indicates that expression of the gene has an activating effect on the signaling pathway, while the blue module indicates an inhibitory effect. (b–g) Relationship between higher and lower expression groups of hub genes and AR signaling pathway activity score. (b) RAD51. (c) BLM. (d) APOE. (e) DTL. (f) RFC2. (g) EXO1. (h–m) Validation of hub genes expression differences in localized Pca (22) and mCRPC (29) based on the GSE32269 dataset. (h) RAD51. (i) BLM. (j) APOE. (k) DTL. (l) RFC2. (m) EXO1. The significance of differences between the two groups was determined using the Wilcoxon test. AR signaling pathway, androgen receptor signaling pathway; PCa, prostate cancer; mCRPC, metastatic CRPC.

Since enzalutamide exerts its inhibitory effect on CRPC mainly through inhibition of AR signaling, we investigated the potential role of hub gene expression levels on the AR signaling pathway’s activity. *RAD51*, *BLM*, *EXO1*, and *RFC2* (Figure 7a) activated the AR signaling pathway (FDR < 0.05), and their expression level correlated with AR pathway activity, while *DTL* and *APOE* had no significant effect on AR pathway activity (Figure 7b–g). Differential expression analysis on the dataset GSE32269 containing primary PCa and mCRPC tissue samples showed that the expression levels of *RAD51*, *BLM*, *DTL*, and *APOE* were significantly upregulated in mCRPC compared to those in primary PCa tissues (FDR < 0.05), while the expression of *EXO1* and *RFC2* were not significantly different (Figure 7h–m).

### 3.7 Validation of the relationship between hub genes and immune infiltration

**Figure 8:**
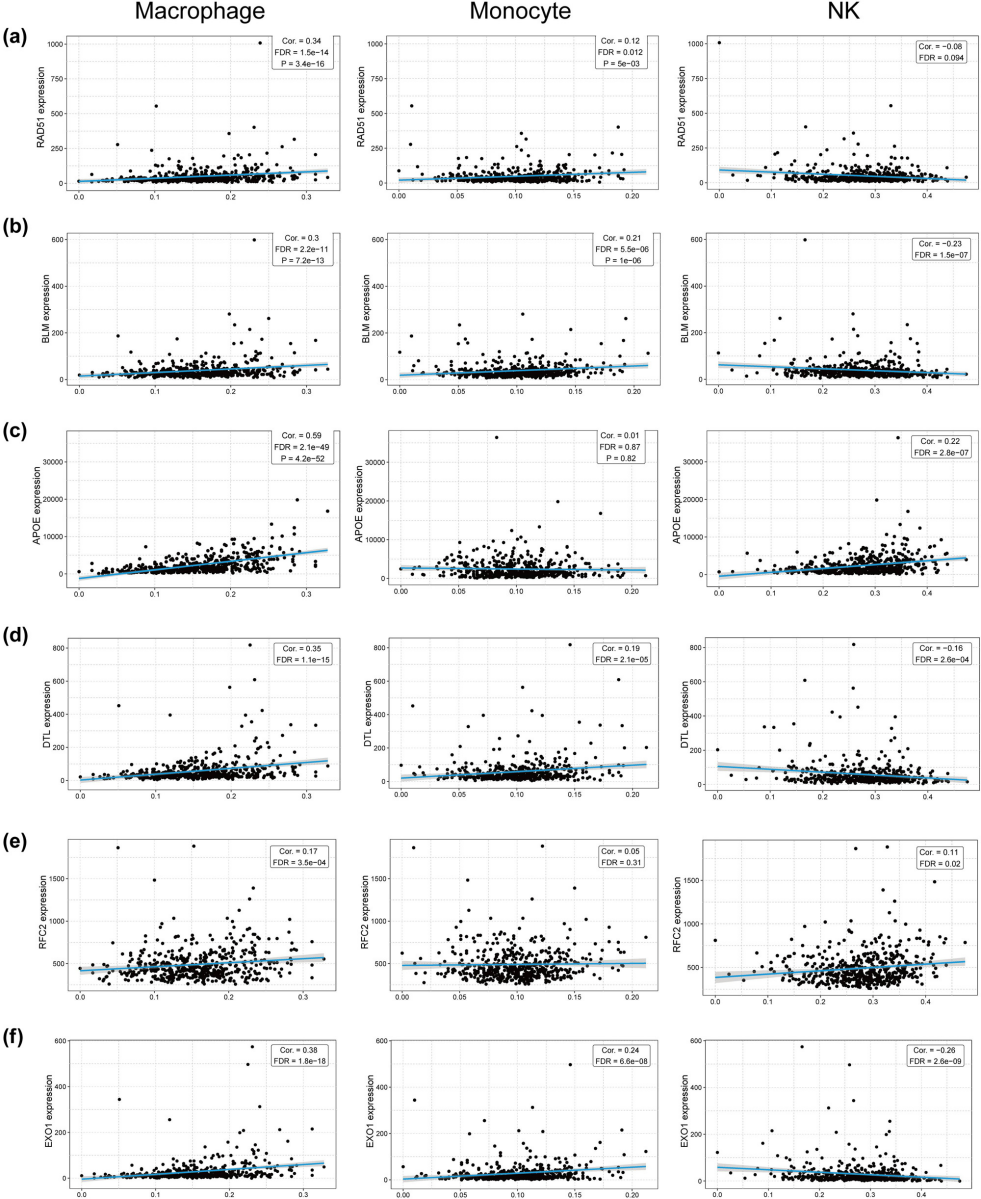
Spearman correlation of hub genes expression in PCa with macrophage, monocyte, and NK cell infiltration. (a) RAD51. (b) BLM. (c) APOE. (d) DTL. (e) RFC2. (f) EXO1. NK, natural killer.

We performed a literature review to investigate if these hub genes mediate CRPC resistance to enzalutamide by affecting the infiltration level of immune cells and found that NK cells, monocytes, and macrophages are associated with enzalutamide resistance [18,19]. In view of this, we analyzed the relationship between the expression of hub genes in PCa and the infiltration levels of NK cells, monocytes, and macrophages. As shown in Figure 8, the expression of *RAD51*, *BLM*, *DTL*, and *EXO1* showed a significant positive correlation with the infiltration levels of monocytes and macrophages, while the expression of *RFC2* and *APOE* only showed a significant positive correlation with the infiltration level of macrophages but not monocytes. In addition, the infiltration level of NK cells showed a significant negative correlation with the expression of *BLM*, *DTL*, and *EXO1*, but not with that of *RAD51*, and a positive correlation with *RFC2* and *APOE* expression.

### 3.8 Relationship between hub gene expression and drug sensitivity

**Figure 9:**
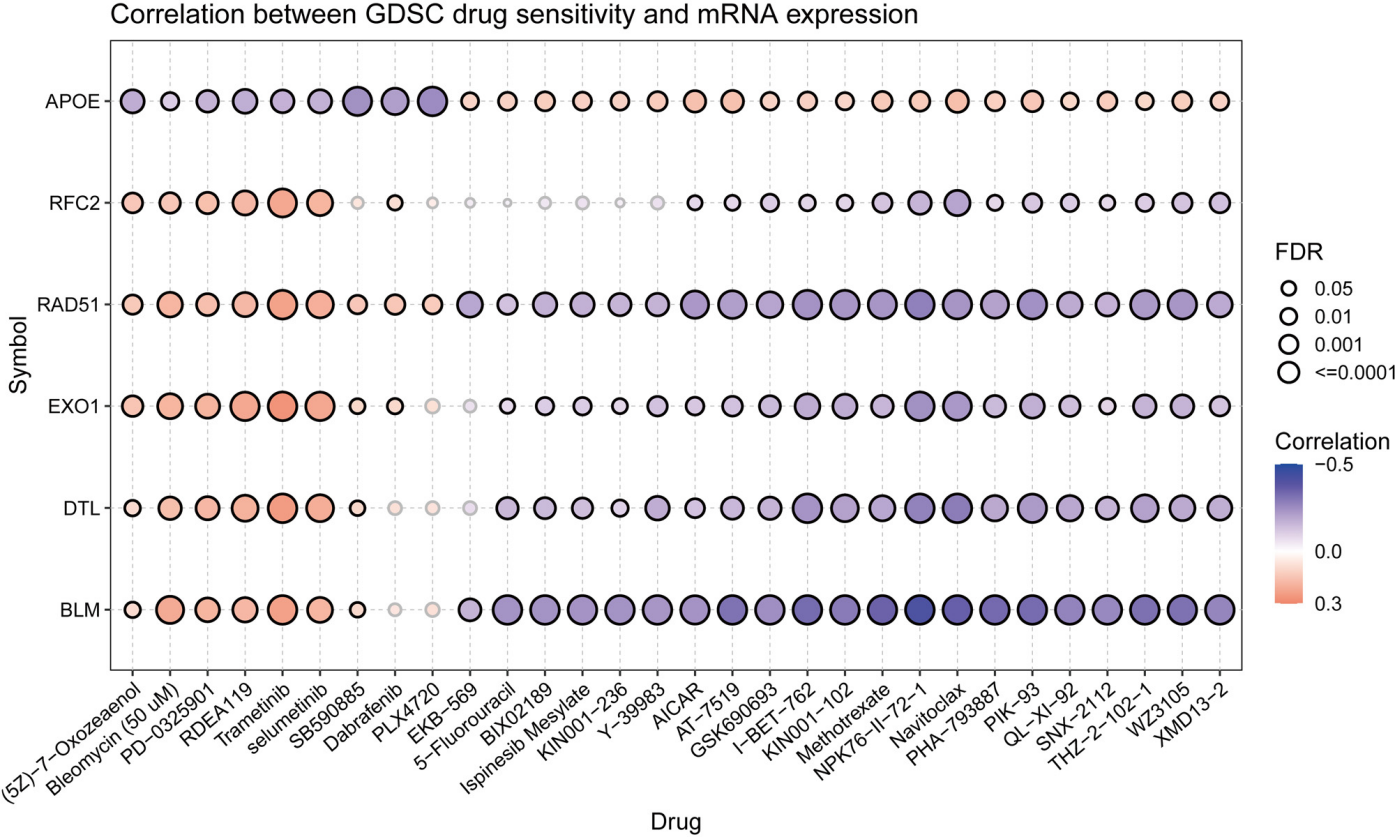
Correlation between GDSC drug (top 30) sensitivity and hub genes expression. The negative correlation indicates that high expression of the gene is sensitive to the drug, and vice versa. GDSC, Genomics of Drug Sensitivity in Cancer.

Based on the data provided by the GDSC database, we analyzed the relationship between the expression levels of hub genes and tumor drug sensitivity (IC50) through the GSCA online data analysis website. A gene’s expression being positively correlated with drug sensitivity indicates drug resistance, and vice versa. The high expression of hub genes, except that of *APOE*, showed a significant negative correlation with the IC50 of Navitoclax and NPK76−II−72−1, while *APOE* expression showed a significant correlation with the IC50 of SB590885, Dabrafenib, and PLX4720 (Figure 9).

### 3.9 Knockdown of *RAD51* inhibits the proliferation and migration of PCa cells and promotes apoptosis

**Figure 10:**
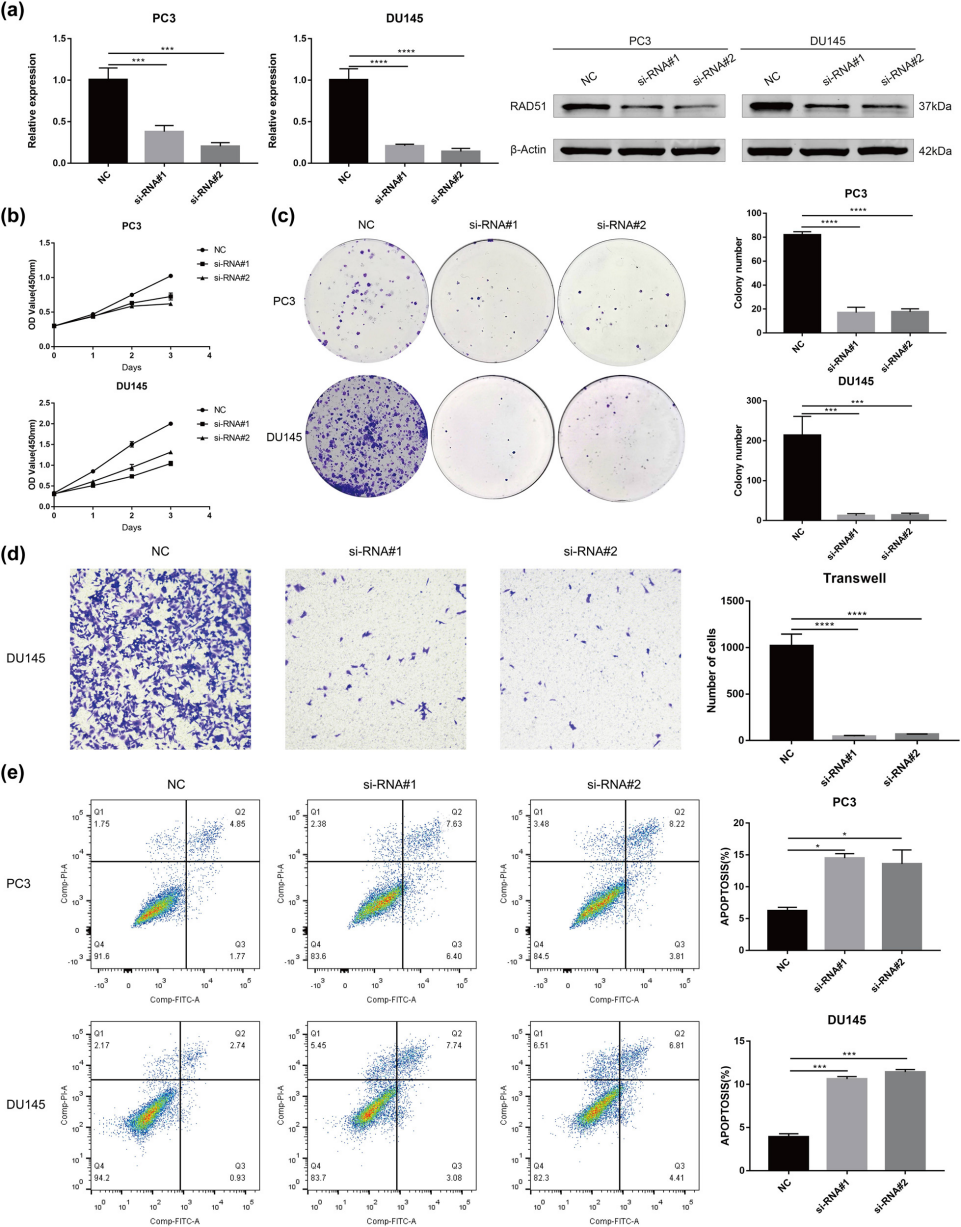
Effect of RAD51 on the proliferation, migration, and apoptosis of PCa cell lines. (a) PC3 and DU145 cells were transfected with siRAD51 to verify the knockdown efficiency of RAD51 at the mRNA and protein expression levels (one-way analysis of variance). (b) CCK-8 detects the proliferation ability of PC3 and DU145 cells after RAD51 knockdown (one-way analysis of variance). (c) Clone formation assay detects the proliferation ability of PC3 and DU145 cells after RAD51 knockdown (one-way analysis of variance). (d) Transwell migration assay detects the proliferation ability of DU145 cell after RAD51 knockdown (100×) (one-way analysis of variance). (e) Flow cytometry assays detect the apoptosis of PC3 and DU145 cells after RAD51 knockdown (one-way analysis of variance). The significance of differences between multiple groups was determined by ANOVA. *P-value < 0.05, **P-value < 0.01, ***P-value < 0.001, ****P-value < 0.0001. PCa, prostate cancer.

**Figure 11:**
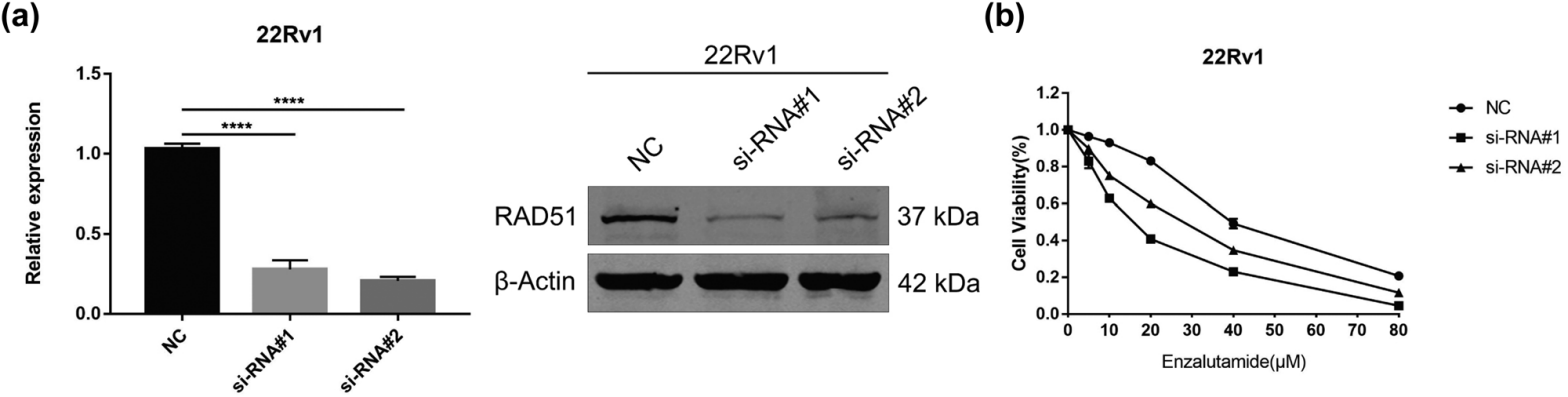
Effect of RAD51 on the proliferation, migration, and apoptosis of PCa cell lines. (a) 22Rv1 cells were transfected with siRAD51 to verify the knockdown efficiency of RAD51 at the mRNA and protein expression levels (one-way analysis of variance). (b) Effect of RAD51 knockdown on the viability of 22Rv1 cells treated with different concentrations of enzalutamide for 48 h as detected using a CCK-8 assay. *P-value < 0.05, **P-value < 0.01, ***P-value < 0.001, ****P-value < 0.0001.

To explore the role of *RAD51* in PCa, we knocked down *RAD51* in PC3, DU145, and 22Rv1 cell lines (Figures 10a and 11a). The CCK-8 and clone formation assays demonstrated that the knockdown of *RAD51* inhibited the proliferative capacity of both PCa cell lines (Figure 10b and c). Moreover, transwell assays showed that knockdown of *RAD51* inhibited the migratory ability of DU145 cells but had no effect on PC3 cells (Figure 10d). Apoptosis assays demonstrated that knockdown of RAD51 promoted apoptosis in two PCa cell lines (Figure 10e). In addition, knockdown of *RAD51* inhibited the proliferation of 22Rv1 cells more significantly than no knockdown of *RAD51* under enzalutamide treatment (Figure 11b).

## 4 Discussion

An increasing number of studies have shown that abnormal expression of certain genes promotes CRPC resistance to enzalutamide [20,21,22]. In the present study, we analyzed six hub genes, *RAD51*, *BLM*, *DTL*, *RFC2*, *APOE*, and *EXO1*, which were significantly correlated with enzalutamide resistance, through bioinformatics analysis from multiple perspectives. The expression of *RAD51*, *BLM*, *RFC2*, and *EXO1* was highly correlated with AR pathway activity, more so in the high-expression group than the low-expression group. Meanwhile, differential analysis of the GSE32269 dataset showed that the expression of *RAD51*, *BLM*, *DTL*, and *APOE* was higher in CRPC tissues than in primary PCa tissues. Curiously, the expression of *RFC2* and *EXO1* in CRPC tissues was not significantly different from that in primary PCa tissues. It is plausible that there was no increase in gene expression, but there may be other mechanisms by which these proteins increase in expression [23]. The AR signaling pathway, a key driver in the uncontrolled growth of malignant PCa cells, is reactivated in CRPC and contributes to CRPC progression [3]. Enzalutamide resistance is associated with the AR signaling pathway [24,25]. Therefore, inhibiting this pathway is a logical strategy for treating CRPC [26]. *RAD51* and *BLM* were shown to be highly expressed in CRPC tissues in our study, and the expression levels were positively correlated with the promotion of AR signaling pathway activity. These results suggest that *RAD51* and *BLM* likely mediate CRPC resistance to enzalutamide by promoting the AR signaling pathway. In addition, we verified that *RAD51* knockdown can inhibit the proliferation and migratory ability of PCa cell lines and promote apoptosis. Not only that, the proliferation of 22Rv1 cells was more significantly inhibited under the treatment with enzalutamide when RAD51 was knocked down than when it was not, which further verified that *RAD51* could promote enzalutamide resistance. This result is similar to the findings of Kohrt et al. [23], who identified 11 genes (*TFAP2C*, *CAD*, *SPDEF*, *EIF6*, *GABRG2*, *CDC37*, *PSMD12*, *COL5A2*, *AR*, *MAP3K11*, and *ACAT1*) whose loss resulted in decreased cell survival in response to enzalutamide. Further experimental validation revealed that three of these genes (*ACAT1*, *MAP3K11*, and *PSMD12*) could serve as proponents of enzalutamide resistance *in vitro* [23]. There is no doubt that these genes may serve as new therapeutic targets for enzalutamide-resistant PCa in the future.

Different cell types and extracellular components of the tumor microenvironment (TME) not only play a pivotal role in tumor progression, but also significantly influence therapeutic efficacy and mediate drug resistance [27]. Immune infiltration in the TME is associated with enzalutamide resistance [28,29]; we found from our literature review that enzalutamide-resistant CRPC cells were better able to recruit NK cells than ordinary PCa cells, and NK cells inhibited enzalutamide resistance in CRPC by targeting AR splice variant 7 (ARv7) [18]. Furthermore, enzalutamide induced neuroendocrine like (NE-like) PCa cells to enhance monocyte recruitment to the tumor region and promoted monocytes toward tumor-associated macrophages (TAMs). Further studies revealed that enzalutamide-treated C4-2 cells expressed higher levels of *NSE* and *CHGA* when co-cultured with NE-like PCa-activated THP-1 cells and sorted into monocytes. In addition, the survival rate of enzalutamide-treated C4-2 cells co-cultured with TAMs was higher than that of enzalutamide-treated cells alone [19]. In summary, NK cells can inhibit enzalutamide resistance in CRPC, while monocytes and macrophages have a facilitative effect on the survival of enzalutamide-resistant CRPC cells. Our results demonstrate that the expression levels of *EXO1*, *BLM*, and *DTL* significantly correlated with the infiltration levels of monocytes, macrophages, and NK cells. The expression levels of *RAD51*, *EXO1*, *BLM*, and *DTL* showed a significant positive correlation with the infiltration levels of monocytes and macrophages. The expression levels of *EXO1*, *BLM*, and *DTL* showed a significant negative correlation with the infiltration levels of NK cells, and finally, the expression levels of *RFC2* and *APOE* were only significantly and positively correlated with the infiltration levels of macrophages. Collectively, our results suggest that the six hub genes may mediate enzalutamide resistance through the different immune cell infiltration pathways.

In our analysis of the relationship between the expression of hub genes and drug sensitivity based on GDSC, high expression of *RAD51*, *BLM*, *DTL*, *RFC2*, and *EXO1* was significantly associated with higher sensitivity to NPK76-II-72-1 and Navitoclax. Studies have demonstrated that CRPC patient-derived conditionally reprogrammed cells (CRCs) are sensitive to Navitoclax [30]. Navitoclax is an inhibitor of BCL-2 protein families, which are often overexpressed in cancer and associated with drug resistance [31]. BCL-2 family members, BCL-XL and MCL1, are involved in enzalutamide resistance through activation of PI3K/AKT signaling, leading to apoptosis evasion [32]. In addition, BCL-2 was found to be overexpressed in enzalutamide-sensitive cell lines, leading to the emergence of enzalutamide resistance. BCL-2 inhibitors suppressed the development of enzalutamide resistance in xenografts [33]. Through inhibition of the BCL-2 family of proteins, Navitoclax can induce apoptosis. Therefore, RAD51, BLM, DTL, RFC2, and EXO1 may be potential targets of Navitoclax. The combination of enzalutamide and Navitoclax may be a promising treatment regimen to counter enzalutamide resistance.

In this study, we identified a total of 1,208 DEGs, including the six key genes mentioned above, associated with enzalutamide resistance by analyzing the datasets GSE151083 and GSE150807. GO enrichment analysis showed that DEGs were mainly enriched in processes such as signal transduction, positive regulation of gene expression, and drug response. KEGG pathway analysis showed that DEGs were involved in the cancer metabolic pathways and pathways in cancer. In recent years, studies have reported that glucose and lipid metabolism are related to enzalutamide resistance [34,35,36].

*RAD51* codes for an enzyme involved in double-stranded DNA break repair [37] is highly expressed in various types of tumors and is associated with poor clinical prognosis [38,39,40]. It can promote the occurrence and development of different tumor types [41,42]. Although *RAD51* has been shown to be overexpressed in aggressive PCa [43], its functional role in PCa has not been investigated. Our study demonstrated that *RAD51* knockdown in PCa cell lines PC3 and DU145 significantly reduced cell proliferation efficiency, promoted apoptosis, and inhibited migration. In addition, it has been well documented that *RAD51* overexpression is associated with tumor resistance to chemotherapy [44,45,46,47]. These results suggest that *RAD51* may be a key gene mediating enzalutamide resistance. Bloom syndrome protein (BLM) is an important enzyme in DNA metabolism [48,49]. Its expression in PCa tissues and PC3 cells is significantly higher than that in non-PCa tissues and benign prostatic hyperplasia cells. Knockdown of *BLM* inhibits PCa cell proliferation and promotes apoptosis, and overexpression can reverse tumor growth inhibition [50,51]. Studies have reported that *EZH2* knockdown has an inhibitory effect on the growth of PCa cells, but overexpression of *BLM* can reverse this effect, and the expression of *BLM* is positively correlated with that of *EZH2* [52]. In addition, it has been shown that *EZH2* expression can inhibit *CCN3* expression, and *CCN3* expression inhibits AR signaling, thereby inhibiting the growth of enzalutamide-resistant PCa cells [53]. This evidence suggests that *BLM* may play an important role in promoting enzalutamide resistance through the *EZH2*-*CCN3* axis. DTL, also known as CDT2, is mainly involved in the regulation of DNA replication and cell cycle [54,55]. It has been well documented that DTL promotes the development of certain cancers [56,57,58]. DTL was shown to be associated with AKT/mTOR activation, while inhibition of the mTOR pathway inhibited the proliferation of enzalutamide-resistant PCa cells, leading to AR or AR-V degradation [59,60]. Therefore, DTL may promote enzalutamide resistance through the AKT/mTOR pathway. *EXO1*, a damage-repair related gene, is associated with the clinical progression, metastasis, and survival prognosis of PCa [61,62]. Moreover, the high expression of *EXO1* is related to the resistance of gastric and ovarian cancers to cisplatin [63,64]. APOE is a protein related to lipid metabolism that is abundantly secreted by hepatocytes and macrophages [65,66]. One study found that enzalutamide-treated C4-2B cells co-cultured with TAMs increased cell survival [19]. In addition, *APOE* has been reported to be highly expressed in a variety of tumor types and to promote cancer progression [67,68,69,70]. Replication factor C is a protein complex consisting of five subunits (RFC1-5) that mediates DNA replication and repair [71]. Forkhead box O1 has been reported to promote temozolomide resistance in gliomas through modulation of RFC2 [72]. Meanwhile, Forkhead box M1 (FoxM1) mediates temozolomide resistance in glioma cells by regulating the expression of *RFC5* [73]. Recently, it has been increasingly reported that the high expression of *RFC2* is associated with the proliferation, migration, and invasion of colorectal cancer and hepatocellular carcinoma. Although all six of the above mentioned hub genes were shown to be associated with cancer progression and we analyzed their relationship with enzalutamide resistance from a bioinformatics perspective, a limitation of the present study is that we did not demonstrate the role of these hub genes in enzalutamide resistance from an enzalutamide resistance cell model. Therefore, their specific role in enzalutamide resistance remains unclear, and future experiments are needed to demonstrate their functions.

## 5 Conclusion

We identified six key genes associated with enzalutamide resistance, *RAD51*, *BLM*, *DTL*, *RFC2*, *APOE*, and *EXO1*, and their role in enzalutamide resistance was validated via bioinformatic approaches in terms of signaling pathways and immune infiltration. In addition, we verified that *RAD51* knockdown can inhibit the proliferation and migratory ability of PCa cell lines and promote apoptosis. Furthermore, the proliferation of 22Rv1 cells was more significantly inhibited with knockdown of *RAD51* than without knockdown of *RAD51* under enzalutamide treatment. *RAD51* is a promotor of enzalutamide resistance. These genes and their protein products may serve as new therapeutic targets for enzalutamide-resistant PCa in the future.
